# Evolution of pharmacogenomic services and implementation of a multi-state pharmacogenomics clinic across a large rural healthcare system

**DOI:** 10.3389/fphar.2023.1274165

**Published:** 2023-11-14

**Authors:** Joel Van Heukelom, Jennifer Morgan, Amanda Massmann, Kristen Jacobsen, Natasha J. Petry, Jordan F. Baye, Samantha Frear, April Schultz

**Affiliations:** ^1^ Sanford Imagenetics, Sanford Health, Sioux Falls, SD, United States; ^2^ Department of Internal Medicine, University of South Dakota School of Medicine, Vermillion, SD, United States; ^3^ Department of Medical Genetics, Sanford Health, Sioux Falls, SD, United States; ^4^ Department of Pharmacy Practice, North Dakota State University, Fargo, ND, United States; ^5^ Department of Pharmacy Practice, South Dakota State University College of Pharmacy and Allied Health Professions, Brookings, SD, United States; ^6^ Translational Software, Inc., Mercer Island, WA, United States

**Keywords:** pharmacogenetics, pharmacogenomics, pharmacist, ambulatory care, health services accessibility, patient education, counseling

## Abstract

**Introduction:** Pharmacogenomics (PGx) aims to maximize drug benefits while minimizing risk of toxicity. Although PGx has proven beneficial in many settings, clinical uptake lags. Lack of clinician confidence and limited availability of PGx testing can deter patients from completing PGx testing. A few novel PGx clinic models have been described as a way to incorporate PGx testing into the standard of care.

**Background:** A PGx clinic was implemented to fill an identified gap in provider availability, confidence, and utilization of PGx across our health system. Through a joint pharmacist and Advanced Practice Provider (APP) collaborative clinic, patients received counseling and PGx medication recommendations both before and after PGx testing. The clinic serves patients both in-person and virtually across four states in the upper Midwest.

**Results:** The majority of patients seen in the PGx clinic during the early months were clinician referred (77%, *n* = 102) with the remainder being self-referred. Patients were, on average, taking two medications with Clinical Pharmacogenetics Implementation Consortium guidelines. Visits were split almost equally between in-person and virtual visits.

**Conclusion:** Herein, we describe the successful implementation of an interdisciplinary PGx clinic to further enhance our PGx program. Throughout the implementation of the PGx clinic we have learned valuable lessons that may be of interest to other implementors. Clinicians were actively engaged in clinic referrals and early adoption of telemedicine was key to the clinic’s early successes.

## 1 Introduction

Pharmacogenomics (PGx) is the study of how an individual’s specific genetic results may impact medication response (Pharmacogenomics. National Institute of General Medical Sciences, 2023). The incorporation of PGx information into the clinical care of patients aims to maximize therapeutic efficacy while minimizing adverse events and toxicities, thereby, improving patient outcomes and decreasing healthcare costs (Jarvis et al., 2022; Morris et al., 2022; van der Wouden et al., 2022). The benefits of PGx guided therapy have been shown in multiple clinical settings (Mega et al., 2010; Cavallari et al., 2018; Greden et al., 2019; Pereira et al., 2020; Oslin et al., 2022; Swen et al., 2023). Recently, PGx testing was shown to significantly decrease rates of medication adverse events by approximately 30% over a 12-week period of time (Swen et al., 2023). PGx implementation efforts are aided by support and guidelines from multiple consortia and working groups. Notable examples include the Clinical Pharmacogenetics Implementation Consortium (CPIC) (Relling and Klein, 2011), the Dutch Pharmacogenetics Working Group (DPWG) (Swen et al., 2008), the United States Food and Drug Administration (FDA, 2023) and the Pharmacogenomics Knowledgebase (PharmGKB) (Hewett et al., 2002).

Although PGx testing has shown benefit in many settings, the broad adoption of PGx testing in clinical practice is lagging (Levy et al., 2020). Reasons for this are multifaceted and include limited prescriber PGx knowledge due to lack of sufficient education, cost, lack of experience in ordering, interpretation and utilization of the results, and clinic visit time constraints (Qureshi et al., 2022). To overcome such barriers, multiple institutions have developed clinics specializing in PGx with varying practice models. Most PGx services discussed in the literature consist of pharmacist consultations within various settings such as executive health visits, outpatient clinic visits and community pharmacies. Additional models include multidisciplinary visits with internal medicine providers, an advanced practice provider (APP), or a genetic counselor (Dunnenberger et al., 2016; Bain et al., 2018; Arwood et al., 2020; Liko et al., 2021; Gammal and Fieg, 2022; Matey et al., 2022; Kehr et al., 2023).

Sanford Health has embraced the motto “Here for All” regardless of location with a dedication to those in rural communities, which have historically had less opportunities to utilize PGx data in their clinical care (Le et al., 2022). In this manuscript, we describe the implementation processes and early successes of implementation at a PGx clinic at Sanford Health.

## 2 Background

### 2.1 Evolution of PGx program prior to clinic implementation

**FIGURE 1:**
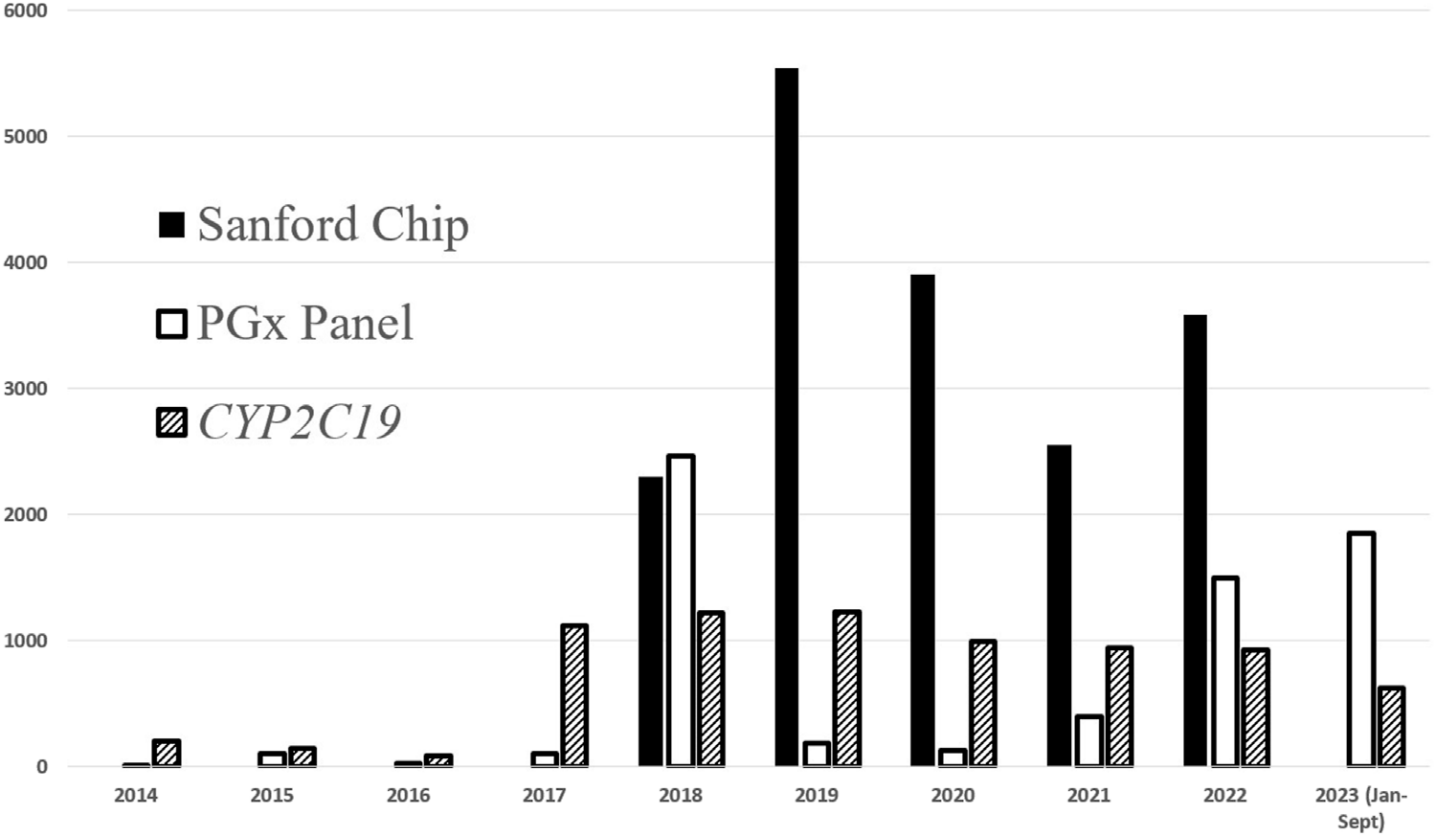
PGx testing volumes completed at Sanford Health. Additional single genes have been ordered at lower frequencies and include: *CYP3A5, CYP2D6, TPMT, DPYD, SLCO1B1, CYP2C9*.

**FIGURE 2:**
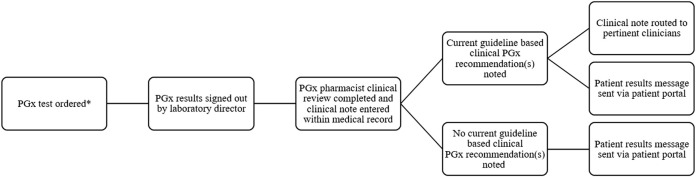
Workflow of pharmacist result review for PGx results review *PGx testing can be ordered by any authorized provider.

In 2014, Sanford Health’s precision medicine initiative, Sanford Imagenetics, was created with the overarching goal of integrating genetic medicine into primary care as described in detail elsewhere (Christensen et al., 2021). The initiative included the creation of a program to offer patients personalized medication management through the integration of PGx into routine care. To date, a total of over 29,000 patients have received PGx testing through various aspects of our program (single gene, PGx panels, and combination preemptive screen) (Figure 1). Our program supports healthcare clinicians with the addition of a comprehensive clinical review provided by a PGx trained clinical pharmacist, considering current and historical medication uses in the context of every patient result and supported with a robust clinical decision support system. Recommendations based on drug-gene interactions and other clinical considerations are shared with the ordering and other relevant physicians and APPs through clinical notes within the electronic medical record (EMR) (Petry et al., 2019). To our knowledge, ours is the only PGx program to perform a clinical review of all pediatric and adult patients when preemptive and reactive clinical testing are completed (Figure 2).

Sanford Health has an in-house medical genetics laboratory, which is Clinical Laboratory Improvement Amendments (CLIA) and College of American Pathology (CAP) accredited. This internal laboratory completes the vast majority of PGx testing ordered within our institution. The current PGx panel testing includes *CYP2C19*, *CYP2D6*, *CYP2C9*, *CYP3A5*, *CYP4F2*, *CYP2C cluster*, *VKORC1*, *SLCO1B1*, *TPMT*, *DPYD*, and *IFNL3* (Table 1). PGx testing is most commonly completed through DNA extracted from whole blood samples within our laboratory. Patient genotyping occurs with TaqMan^®^ qualitative polymerase chain reaction assays (ThermoFisher Scientifics; Waltham, MA) utilizing Fluidigm SNP Dynamic Array (Standard Biotools; South San Francisco, CA). Additionally, assessment of *CYP2D6* copy number is performed by droplet digital PCR (Bio-Rad; Hercules, CA). An expanded panel (allele coverage and additional genes) is underway with plans to implement in late 2023. The expanded panel will utilize targeted next-generation sequencing technology. The PGx panel has evolved with evidence updates as well as technical feasibility within the medical genetics laboratory. As the panel expands, multiple references are utilized to assist in the clinical validity of the panel such as consensus statements from the Association for Molecular Pathology, CPIC, CAP, DPWG, PharmGKB, European Society for Pharmacogenomics and Personalized Therapy and Pharmacogenomics Knowledgebase (Pratt et al., 2018; Pratt et al., 2019; Pratt et al., 2020; Pratt et al., 2021; Pratt et al., 2022; Pratt et al., 2023).

**TABLE 1:** Current PGx panel genes, alleles and clinical decision support.

Gene tested	Alleles tested	Medications/Classes with clinical decision support
*CYP2C19*	*2, *3, *4A, *4B, *5, *6, *7, *8, *17	Clopidogrel
		Proton Pump Inhibitors
		Tricyclic Antidepressants
		Selective Serotonin Reuptake Inhibitors
		Voriconazole
*CYP2D6*	*2, *3, *4, *4M, *6, *9, *10, *17, *29, *41, *5 (gene deletion), XN (gene duplication)	Tricyclic Antidepressants
		Selective Serotonin Reuptake Inhibitors
		Serotonin and Norepinephrine Reuptake Inhibitors
		Serotonin Modulators
		Opioids
		Atomoxetine
		Ondansetron
		Aripiprazole
		Metoclopramide
		Brexpiprazole
		Iloperidone
		Pimozide
		Eliglustat
		Pitolisant
		Deutetrabenazine
		Tetrabenazine
		Valbenazine
*CYP2C9*	*2, *3, *5, *6, *8, *11	Warfarin
		Non Steroidal Anti-inflammatories
		Phenytoin/Fosphenytoin
		Statins
		Siponimod
*CYP3A5*	*3, *6, *7	Tacrolimus
*CYP2c cluster*	g.9640552G>A	Warfarin
*CYP4F2*	c.1297G>A	Warfarin
*VKORC1*	1639G>A	Warfarin
*DPYD*	c.1905 + 1G>A, c.1679T>G, c.2846A>T	Fluoropyrimidines
*TPMT*	*2, *3A, *3B, *3C, *4	Thiopurines
*SLCO1B1*	*5, *15, *37	Statins
*IFNL3*	rs12979860	PEG Interferon-α–Based Regimens

Current panel expansion planned to include greater allele coverage for current genes as well as increased number of genes (*CYP2B6, NUDT15, UGT1A1*).

PGx results are integrated into the EMR through a Health Level Seven (HL7) interface. Through incorporation of PGx results as structured or discrete data in the EMR, clinical decision support systems have been constructed to provide real-time guidance for clinicians. The PGx team has devised eighty-eight alerts for drug-gene interactions and five alerts for drug-gene-disease interactions.

**FIGURE 3:**
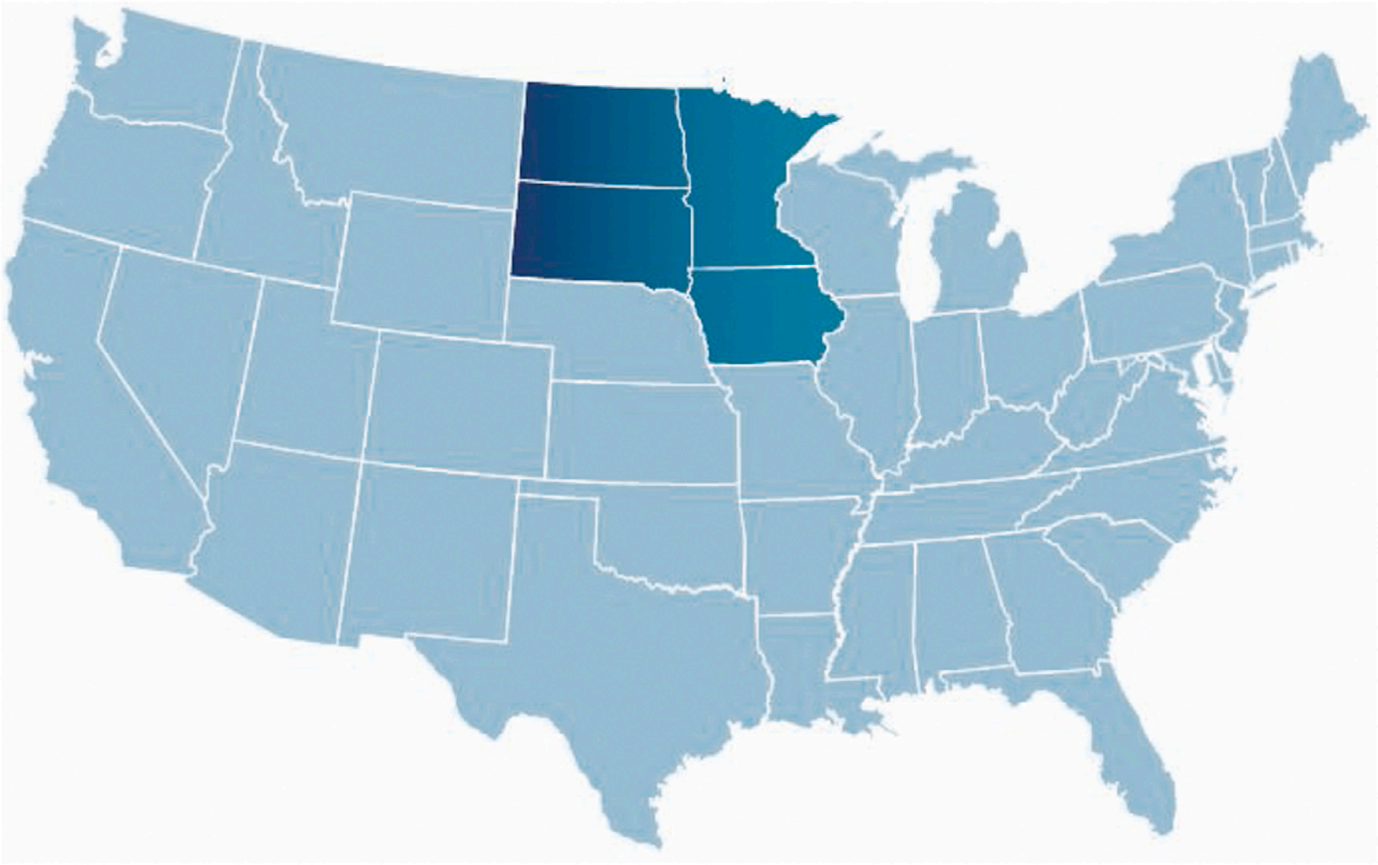
Geographic region served by the Sanford Imagenetics Pharmacogenomics (PGx) Clinic.

**FIGURE 4:**
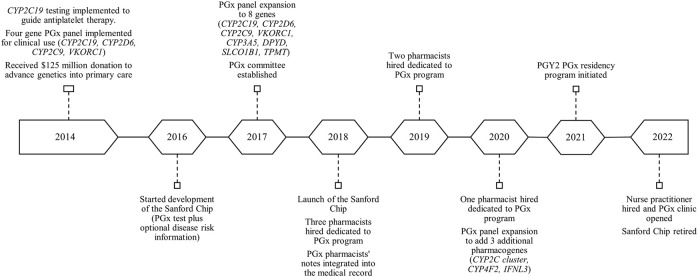
Timeline of the evolution of PGx services offered at Sanford Health.

Our PGx program has a wide geographical reach. Sanford Health spans across multiple states in the upper Midwest and includes 46 medical centers, 222 clinic locations, and employs approximately 1,500 physicians and 1,300 APPs. Figure 3 outlines the geographical service area of the Sanford PGx Clinic spanning nearly 250,000 square miles. As an enterprise-wide department, Sanford Imagenetics, serves a large, predominantly rural, patient population. The PGx pharmacy team is comprised of six experienced clinical pharmacists and an ASHP-accredited PGY2 Clinical PGx resident position. The PGx program has evolved throughout the years and PGx volumes have increased secondary to institutional emphasis and integrating genomic information into primary care (Figure 4). Initial testing was championed by physicians in both internal medicine with PGx training as well as an interventional cardiologist. Initial clinical decision support content was supported through a contract pharmacist. In 2018 with the launch of a system wide emphasis on PGx testing, three internal pharmacists were hired with additional pharmacists hired based on high demand for services.

As early adopters of PGx integration, we empowered front line physicians and APPs to return PGx results to patients following clinical pharmacists review. Internal analysis of previous institutional educational efforts that aimed to increase clinician uptake of PGx revealed a lack of familiarity and comfort in ordering PGx testing and uncertainty of clinical utility by some clinicians (Hajek et al., 2022; Preys et al., 2023). To fill this gap, a strategic initiative to implement a PGx clinic was developed. To achieve our goal of incorporating PGx into routine care for all patients across the Sanford Health system, it was imperative to offer virtual care services. Given the novelty of these clinics we initiated a research protocol at the onset of seeing patients. Here we report the creation of the clinic, research aspects, referral workflows, educational offerings, appointment processes, and lessons learned through caring for the first 102 patients seen in our PGx clinic.

## 3 Clinic development and implementation

Key considerations in the development of the Sanford Imagenetics PGx clinic were the operational model, patient and staff education, and research. The PGx clinic is located within the Sanford Imagenetics building in Sioux Falls, South Dakota, which houses other service lines including the Sanford Medical Genetics Laboratory, Internal Medicine Clinic, Medical Genetics Clinic, Genetic Counseling services, PGx pharmacy team, phlebotomy services, and Imagenetics leadership. The availability of these service lines in combination with the in-house PGx testing by the Sanford Medical Genetics Laboratory made this an ideal location for the clinic. Multiple specialties are housed within the same building; however, each specialty has its own physical space for patient visits. At the time of PGx clinic implementation planning, Medical Genetics planned to expand their service line through incorporation of an APP specializing in genetics. Following a year delay secondary to the COVID-19 pandemic, the clinic began seeing patients in April of 2022. Initial projections were focused on seeing a majority of patients who had already completed testing given the vast number of patients who had PGx testing completed prior to the opening of the PGx clinic.

### 3.1 PGx clinic visit structure

Our clinical service model is a joint visit type between a clinical pharmacist and an APP. In collaboration, both deliver patient education, obtain research consent surrounding the PGx clinic research, and answer patient questions during patient visits. Each discipline brings a unique skill set. The pharmacist collects a detailed medication history while the APP reviews medical history and completes a physical examination. The APP and clinical pharmacist provide therapeutic recommendations to patients’ relevant providers. PGx recommendations are augmented with general medication management, medication education, and disease state education. We believe it is important to discuss how their results will assist in medication management throughout their lifetime and are guided by clinical decision support within our health system. It is important for patients to understand certain limitations within clinical decision support, such as issues with phenoconversion. Accordingly, clinicians are educated on how PGx results should be used in coordination with other clinical factors, such as drug interactions and changes in disease state(s).

The PGx clinic visit structure varies depending on multiple factors, such as patient characteristics, needs, and the visit type. During in-person visits, the pharmacist and APP often see the patient sequentially and are not both present during the entirety of the visit. However, for patients with complex or lengthy medication regimens, both the APP and pharmacist may be present concurrently to decrease redundancy in questions. Due to limitations of the telehealth platform, the APP must initiate the patient visit. Typically, both the pharmacist and APP are present during the entirety of telehealth visits. All visits are scheduled for 1 h in length.

PGx clinic visits are billed under medication management, with reimbursement based on APP time. Given the novelty of this clinic within our health system, we created reports to track reimbursement for clinic visits to ensure creation of a sustainable model. We will report these results in a future manuscript. Pharmacist time is not currently reimbursable for most services within our footprint and is covered by operational funds within our precision medicine initiative.

**FIGURE 5:**
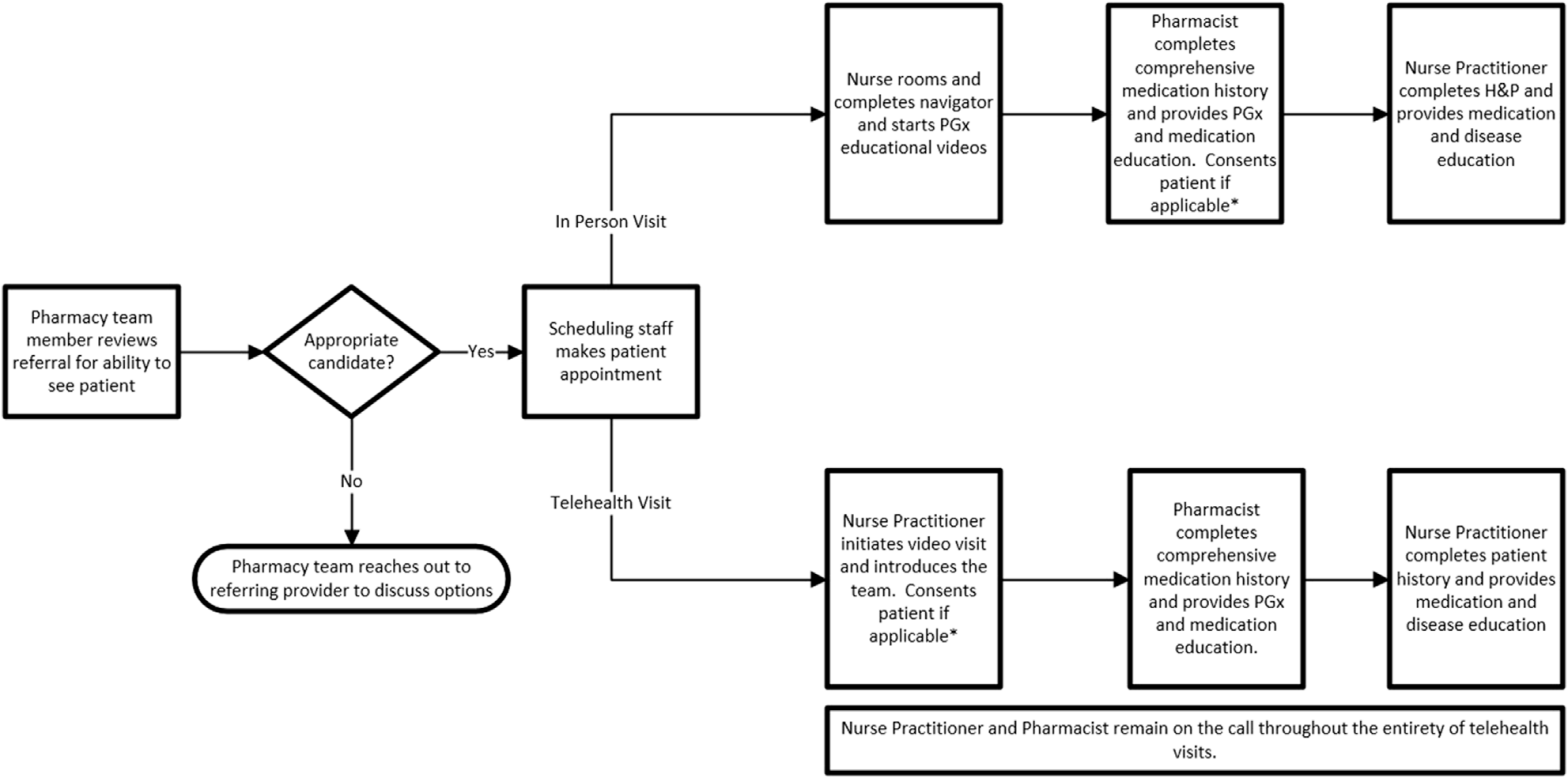
PGx Clinic Workflow. * Patients consented during first clinic visit, therefore, if patients have pre and post-test visits they will not be approached for consent during their post-test visit. Exclusions: Pediatric patients; clear documentation of an indication we are unable to provide information on (ex: allergy testing); referrals for disease predisposition H&P, History and physical; APP, Advanced Practice Provider.

We offer visits prior to PGx testing (pre-test) and after testing (post-test). Patients can be seen virtually or in-person at the clinic in Sioux Falls, South Dakota. Depending on the needs of the patient or referring provider, one or two visits are utilized (Figure 5). Within our healthcare system, PGx testing is orderable by all providers at their discretion, therefore, there is not a requirement for pre- or post-test visits to obtain PGx testing. There are several ways a patient may obtain and review PGx testing. Providers may order testing and review results with their patients, order testing and refer to the PGx clinic for review of results (post-test) or refer to the PGx clinic to discuss PGx testing and defer the ordering of testing to our clinic based on shared decision making during the clinic visit (pre-test). If testing is ordered during a pre-test visit, patients are scheduled to return to the PGx clinic for results review (post-test). Given the numerous scenarios possible, at times we see patients only for a pre-test visit (no PGx testing ordered during visit), only for a post-test visit, or both a pre- and post-test visit.

The primary focus of pre-test visits is informed consent of PGx testing. Through discussions of benefits, limitations, and cost considerations (variable insurance coverage), we strive to provide patients with realistic expectations of PGx testing. Post-test visits provide patients with an in-depth explanation of their results and implications for current and future medications. For patients completing two visits, appointments are scheduled at least 2 weeks apart to allow for results to be returned. When PGx testing is ordered, it is routinely completed through our in-house Sanford Medical Genetics Laboratory. Turnaround time for lab testing is 7–10 days from when the sample is received in the laboratory. Given our broad geographical reach, some blood samples must be couriered to our laboratory.

### 3.2 PGx clinic educational efforts

Initial clinician PGx clinic educational efforts were targeted toward primary care providers, specifically internal medicine and family medicine clinics in regions of the Sanford Health system in close proximity to the physical clinic. Ultimately, educational efforts were extended across the health system as virtual care visits became available. Staff were introduced to the newly available PGx clinic services through in-services that included information on the clinic model, appointments, referrals, and resources for clinical PGx questions. Basic PGx education and indications for testing were also reviewed as a refresher from previous education. The PGx team is available as a resource for staff via phone, email, and secure messaging within the EMR.

### 3.3 PGx clinic referral process

Referrals are either clinician or patient initiated based on either clinician discretion and/or patient interest. The clinic accepts both internal and external referrals. Clinician referrals are placed within the EMR for those within the health system; external referrals are faxed. All referrals are screened by a member of the PGx pharmacy team to ensure the appropriate referral specialty was selected (PGx vs. Medical Genetics, vs. Genetic Counseling). Patient initiated referrals are accepted via multiple pathways, most typically patients reach out via phone to our clinic. We see patients with questions regarding the utility of PGx testing, how PGx testing could assist in their care, or result interpretation of both internal and external PGx data. At this time, we do not integrate external results into our EMR as discrete results, therefore, external results are accessible only within the scanned section of the medical record.

### 3.4 Patient educational efforts

Patient education materials (printed, audio, and visual) were developed for use before, during, and after the PGx clinic visit. Clinic brochures were placed in waiting areas within primary care clinics to generate awareness of the benefits of testing and availability of the clinic. At the beginning of the in-person appointment, patients are shown an educational video based on visit type (basics of PGx or terminology used to help explain results). Patients are also given handouts with basic PGx information and a copy of their test results with a color-coded guide of medications with PGx guidance. Handouts utilized include medication specific information sheets that discuss the impact of genetic variants of predicted medication response, an educational handout specifically geared towards medications for mental health, an overview of PGx and how medications are metabolized leading to interpatient variability in response, and patient specific genetic results and impacted medications. PGx results are also available within the patient portal of the EMR in two formats, both the lab data (genotype, phenotype, activity score) as well as through medication specific Genomic Indicators (Epic Systems Corporation, Verona, WI). Educational material is also available on the health system’s website.

### 3.5 PGx clinic research

During the planning process, the team identified contribution to the literature as a goal and incorporated a research consent into the clinic workflow. Patients are approached for research consent during their first clinic visit. The primary focuses of the research projects are patient literacy and satisfaction with their PGx clinic visits, health outcomes, and economic outcomes. The protocol was approved by the Sanford Health Institutional Review Board (STUDY00002861). Patients are offered to consent to partake in outcomes research related to the PGx clinic, specifically following medical records, financial information, and permission to contact the patient via personal email with survey links. Patients’ involvement in our research endeavors, at this time, only extend to requesting one survey following their PGx clinic visit(s). Patient reported data via surveys will be collected first followed by outcomes data over time. This paper aims to report initial demographics of clinic patients with survey and outcomes data to be reported in a future manuscript.

## 4 Initial results of PGx clinic

During the first 11 months of operation of our PGx clinic, we completed 131 visits for 102 patients, 64 (49%) pre-test and 67 (51%) post-test visits. Twenty-nine patients completed both pre- and post-test visits. Of the 64 pre-test visits, 49 (77%) patients had PGx testing ordered during the visit (11 gene panel offered through Sanford Medical Genetics Laboratory). All patients with PGx testing results had at least one variant on their results. The most common reasons for foregoing PGx testing were financial considerations as well as lack of current PGx guidance on specific medications of interest. Sixty-three visits were conducted virtually for patients in states outside the primary location of the clinic (i.e., Minnesota, Iowa, North Dakota). Month to month visit growth was variable, ranging from 75% decrease to 450% increase (average 95% growth). Trends have been noted with increases following provider educational efforts, however given initial results and anecdotal feedback, this is hypothesis generating at this time.

Eighty-eight out of 102 patients consented to participate in prospective research, whose demographics are described in Table 2. Mean patient age was 51 years and were predominantly female (75%). On average patients were receiving two medications for which CPIC guidance was available at the time of their first PGx clinic visit. Patients without current CPIC medications were often seeking treatment and guidance for CPIC related medication(s) prior to initiation of therapy. Referrals were more likely to be physician or APP (77%) versus patient initiated. Most referrals were from primary care physicians (family medicine and internal medicine); however, we did receive referrals from providers specializing in obstetrics and gynecology (n = 4), genetic counseling (n = 3), endocrinology (n = 2), behavioral health (n = 1), cardiology (n = 1), medical genetics (n = 1), neurology (n = 1), and orthopedic surgery (n = 1). All patients with PGx results had at least one variant in a pharmacogene. This finding highlights the opportunity for medication optimization across the patient population and specialties. Depending on the time of PGx testing, patients may have had a PGx panel consisting of 8 genes (*CYP2C9, CYP2C19*, *CYP2D6*, *CYP3A5*, *DPYD*, *SLCO1B1*, *TPMT*, and *VKORC1*) or 11 genes (addition of *CYP2C cluster*, *CYP4F2*, and *IFNL3*).

**TABLE 2:** PGx clinic patient characteristics.

Characteristics	N = 88
Age [Avg. (range)]	51 (19–84)
Sex	
Female (n, %)	66 (75%)
Male (n, %)	22 (25%)
CPIC Medications [Avg. (range)] ^	1.98 (0–4)
PGx Variant Present (n, %) *	75 (100%)
Referral Type	
Clinician	68 (77%)
Self	20 (23%)

^Based on medication list at time of first PGx, clinic visit.

*Does not equal number of participants as not all patients chose to have testing completed or have test results pending; based on either 8 gene or 11 gene PGx, panel testing.

## 5 Reflection

### 5.1 Successes

The utilization of technology to serve a large geographical area with a small team has been one of our greatest successes. We encountered similar barriers to PGx implementation as described in the literature with the additional challenge of providing services for patients across a multi-state footprint (Qureshi et al., 2022). We chose to offer both in-person and virtual visits to increase access for patients not located within close geographical proximity to the clinic. The wide-scale implementation of virtual care visits required additional technological and financial resources. The existing virtual infrastructure of the health system, which was greatly expanded during the COVID-19 pandemic, was utilized by the PGx clinic. The APP and PGx pharmacists obtained multi-state licensure. Additionally, consideration was given to the limitations of virtual visits, particularly the inability to perform physical exams; however, we did not believe this to be a strong detractor from the services provided by the PGx clinic. Despite multiple challenges, we have successfully completed virtual visits across four states, providing care for patients that would not have otherwise been able to access our PGx clinic.

Our clinic model has proved effective to date as it allows for a billable service, which will be reported on in a future publication, and aligns with our preferred patient experience, existing infrastructure, and supporting literature (Dunnenberger et al., 2016). Our APP has a shared position between the PGx clinical team and Medical Genetics which has proven many benefits. Sharing staffing costs made the model feasible. Additionally, APP onboarding and ongoing collaboration is supported by multiple genetics-focused specialties within the same clinical space (PGx, Medical Genetics, and Genetic Counseling). Additionally, a dual-trained clinician can provide patient education on genetic disease predisposition in addition to PGx. This is useful as approximately 18,000 patients within the health system have had combination preemptive screening which included both PGx and disease predisposition information (Christensen et al., 2021). The APP also identifies patients who would benefit from a referral to Medical Genetics or qualifies for genetic testing and assists in the management of these patients.

Initial data suggests uptake by clinicians is promising as most patients were being referred to our clinic by a provider. Given the novelty of this service, we knew education was paramount to success. While referring clinicians were largely primary care providers, we did see interest from a wide variety of specialists.

The number of pre-test visits exceeded projections. We anticipated the majority of clinic visits would be following testing; however, data revealed the amount of pre-versus post-test visits were almost equal. We believe this demonstrates the desire of patients to understand PGx implications prior to obtaining PGx testing as well as clinicians need to offload these lengthy discussions necessary for proper informed consent. Furthermore, the high percentage of tests ordered during pre-test visits shows patients often want PGx testing when the benefits and limitations are thoroughly discussed.

### 5.2 Barriers and challenges

As with any new service line implementation, ours was not without challenges. The process improvements are detailed in Table 3. The primary strategies were refining workflows for scheduling and mailing materials and providing education to clinicians and patients. Ensuring patients had reliable internet connectivity and correct patient location reduced service disruption. Test results were mailed to patients prior to the post-test visit to facilitate a meaningful discussion of complex results. Patients were educated on lab turnaround times and virtual visit follow-ups were scheduled 2 weeks out, which subsequently was changed to 3 weeks to allow more time for patient lab draws and PGx sample processing. External PGx results are scanned into our medical records thereby negating clinical decision support capabilities; patient education on this limitation was an emphasis during clinic visits with patients having external results.

**TABLE 3:** Key lessons learned throughout our implementation process.

Barrier/Challenge	Process improvement
Internet connection during virtual visits	Scripted instructions sent to patients via patient portal of EMR to test connectivity prior to appointment
Patient located outside of virtual care state	Patients educated when scheduling appointment about location during visit in South Dakota, North Dakota, Minnesota, or Iowa
Lack of visual material during virtual visits	Algorithm developed for materials to be mailed to patient prior to post-test visit (PGx results and educational printouts)
Delays in blood draw with virtual visits Transportation time for samples to arrive at our laboratory	Adjusted scheduling post-test visit from 2 to 3 weeks for video visits
	Increased patient education on the turnaround time for test results
Provider referral confusion	Increased provider education through clinic in-services and direct communication
Non PGx related referrals	Real time provider or patient education
	Collaboration with Medical Genetics colleagues
PGx clinic volumes	Continued outreach to increase awareness of the new service line
External PGx results	Patient education on lack of clinical decision support with external results
	Patient education and process to address drug-gene interactions that may result in serious harm (*CYP2C19*-clopidogrel, *DPYD*-Fluorouracil, etc.)
Standardizing pharmacist workflow and educational content	Regular team meetings to discuss best practices with incorporation into visits
Electronic referral mapping	Report developed to review referrals on a weekly basis to ensure all referrals being routed appropriately

The referral process remains an area of focus for improvement. Provider educational in-services and real time communication on ordering testing versus a clinic referral has been beneficial, however, incorrect referrals continue to be placed. Referrals for genetic counseling and geneticists are redirected to the appropriate provider. Initially electronic referrals were not routed appropriately which resulted in scheduling delays and a loss of appointments which could have impacted future referrals. The team now utilizes a weekly report to track electronic provider referrals. The one to two visit model necessitates a continual stream of new patient referrals; continued outreach to clinics has been successful in increasing awareness of the service line.

### 5.3 Future directions

As we look to the future of our PGx clinic, we highlight key focus areas:

• Examine research outcomes data and utilize findings to optimize currently available services and clinical care. The first phase of future research endeavors will focus on patient reported satisfaction and PGx literacy through surveys followed by reporting the clinical and economic impact of the PGx clinic via data derived from the medical record.
• Establish outreach clinics, to extend and enhance PGx services to those outside of our immediate in-person service area.
• Expand virtual care capabilities to additional states.
• Increase awareness of the utility of this service line and PGx testing as a medication safety strategy.
• Continue to share findings for others seeking to implement PGx clinical services.
• Examine feasibility of saliva samples as opposed to blood samples for routine use.

## 6 Discussion

Integrating a PGx clinic within a large health system is feasible but requires significant efforts to ensure clinicians have access to resources and patients have access to care. Early integration of virtual care visits was key to ensure access to care across a large rural system. Given the volume of visits amongst the two visit types, pre-test versus post-test, were similar, it may suggest patients and providers are seeking to gain a better understanding of benefits and limitations of PGx testing. Joint appointments with a pharmacist and APP allow for interdisciplinary discussion and additional face-to-face time with the patient beyond typical office visits. The model we adopted most closely matches the PGx clinic reported utilizing a joint visit style with an APP (Dunnenberger et al., 2016), with the key difference being the addition of a telehealth option for patients residing in rural geographic areas. The framework described within this paper can be modified to develop PGx clinic models tailored to the needs and resources of other healthcare systems.

## 7 Conclusion

A multidisciplinary team has been able to serve a vast geographical region to support PGx implementation in a multistate health system that was an early adopter of integrating genomic information into the routine care of their patients.

## Data Availability

The original contributions presented in the study are included in the article/Supplementary Material, further inquiries can be directed to the corresponding author.
